# User involvement in a Danish project on the empowerment of cancer patients – experiences and early recommendations for further practice

**DOI:** 10.1186/s40900-018-0105-3

**Published:** 2018-08-13

**Authors:** Clara R. Jørgensen, Nanna B. Eskildsen, Anna T. Johnsen

**Affiliations:** 1 Department for Disability Inclusion and Special Needs, School of Education, University of Birmingham, B15 2TT, Edgbaston, Birmingham UK; 2 Department of Psychology, University of Southern Denmark, Campusvej 55, DK-5230 Odense, Denmark; 3 Department of Palliative Medicine, Bispebjerg Hospital, DK-2400 Copenhagen, Denmark

**Keywords:** User-involvement, Patient and public involvement, Patient engagement, Cancer, Empowerment, Denmark

## Abstract

**Background:**

This paper reports on the process of involving former and current cancer patients and carers as co-researchers in a Danish mixed methods research project on patient empowerment of cancer patients in follow up (The Empowerment study 2015–2019). User-Involvement in health care research is a relatively new practice in Denmark and the Empowerment project was one of the first to systematically involve patients and carers in its research design, conduct and reporting. The paper has two aims: first, it provides a detailed account of the process of involving co-researchers in the Empowerment project and second, it presents findings from a workshop held with academic researchers and co-researchers on the project to discuss their experiences and recommendations for user-involvement in the Danish context.

**Methods:**

The Empowerment project adopted a consultative and collaborative approach to user involvement and co-researchers were involved from the early stages and all through the project. Users gave feedback on the proposal, helped develop project documents and research tools, acted as peer interviewers in qualitative interviews, participated in data analysis and development of questionnaires, and co-authored journal articles. The workshop held with the academic researchers and co-researchers consisted of two parallel focus groups and a joint group discussion, following an interactive and informal format to facilitate discussion and exchange of ideas.

**Findings:**

The focus group resulted in eleven recommendations for the further development of user-involvement in Denmark. Key issues encountered were the general lack of guidelines on user-involvement in the Danish context and the need for more organisational support. Particular issues, such as payment, recruitment and training, need to be carefully considered within individual projects and within the national context in which projects are carried out.

**Conclusion:**

The paper adds to the current very limited knowledge base on user-involvement in the Danish context and provides a set of early recommendations for the further development of the practice in Danish Health Research. User-involvement needs to be developed with consideration to the local context, but common challenges also emphasise the usefulness of cross-country comparisons and knowledge exchange.

**Electronic supplementary material:**

The online version of this article (10.1186/s40900-018-0105-3) contains supplementary material, which is available to authorized users.

## Plain English Summary

This paper describes the involvement of former and current cancer patients in a Danish research project on patient empowerment of cancer patients in follow up (The Empowerment study). User-Involvement in health care research is a relatively new phenomenon in Denmark and not much is written about the practice in the Danish context. Documenting such practices is therefore of importance to provide Danish researchers with examples they can draw on and to enable comparisons with other countries that may have different traditions, approaches and contexts for involvement.

The paper has two aims: first, it describes the process of involving former and current cancer patients as co-researchers in the Empowerment study and gives a detailed account of the methods used, including recruitment, training and activities. Second, it presents findings from a workshop with academic researchers and co-researchers in the study. The aim of the workshop was to explore the experiences of both academic researchers and co-researchers and through this, to provide early recommendations for the further development of user-involvement in the Danish context.

Eleven possible recommendations were derived from the analysis of the workshop. These highlight the need for more support and guidance for Danish researchers in how to involve service users. They also illustrate the importance of carefully considering recruitment, training and payments to ensure that it is done in a way that is appropriate in the local context and enables a diverse group of service users to participate.

## Introduction

The involvement of service users and carers in health and social care research is becoming increasingly common internationally [1–3]. In the UK, a wealth of initiatives, organisations and networks to facilitate and strengthen the involvement of service users and carers in research have developed within higher education institutions (e.g. Comensus at the University of Central Lancashire, PenPIG at the University of Exeter and UNTRAP at the University of Warwick), and a national organisation for involvement (INVOLVE) was set up in 1996, producing a range of guidelines and resources e.g. [4–6].

In other countries, such as Denmark, the formalised process of involving service users, patients or other members of the public in health care research is rarer. Projects are seldom evaluated [7] and there are currently few examples [8] and no consistent guidelines to draw on. Documenting and reporting on user-involvement in Danish health research is therefore of importance both nationally, where it can help provide examples of good practice, and internationally, as important lessons can be learned from comparing countries with different traditions of participation, inclusion and health delivery, as well as different degrees of health and social inequalities.

In this paper, we present and discuss experiences of involving former and current cancer patients in a Danish mixed methods research project on patient empowerment of cancer patients in follow up (The Empowerment study 2015–2019). The study is funded by the Danish Cancer Society, who had required user-involvement in the research project as part of the call for funding. The overall aims of the project were to explore the experiences of empowerment of cancer patients in follow up and develop a patient reported outcome measure (PROM) assessing levels of empowerment within this group (for a description of the study and its aims see [9, 10]). As one of the first health care studies in Denmark, the project involved service users in the research design, conduct and reporting from its beginning. As the project evolved, we experienced a large interest in this particular element of the study, and consequently, the description of the process and impact of involving service users became an increasingly prominent part of the study.

In the development of the project, the team drew on experiences from the UK, where one of the project researchers was based and had worked extensively with user-involvement prior to commencing the project. However, in our work and discussions with the patient representative group in the Empowerment study, we encountered some subtle but important differences between the UK and Danish context. These showed that even though much can be gained from using expertise from one setting (with more experience) in another (with less experience), the practice of user-involvement and any recommendations as to how it should be organised need to be developed locally or adapted to the local setting. Acknowledging this point and the lack of Danish guidelines on user-involvement, the study team set up a meeting with the involved (patient) co-researchers and academic researchers on the project to discuss our experiences and to develop an initial set of recommendations for the further development of user-involvement in the Danish context.

This paper has two main aims. First, it describes the methods of involving service users in the empowerment study to provide a background for the later discussion of practice. This part responds to calls by Staniszewska et al. [11, 12] for more thorough and consistent reporting of user-involvement and aims to fill some of the gaps in documentation of Danish user-involvement (for a GRIPP2 checklist, see Additional file 1). Second, we present findings from the meeting held with the co-researchers and academic researchers to discuss views and experiences of user-involvement in the Empowerment study, and provide eleven recommendations for the further development of user-involvement in Danish health research, derived from the meeting.

### Defining and conceptualising user-involvement in the empowerment study

User-involvement’ and Patient and Public Involvement (PPI) are the terms most commonly used in the UK context to describe the process of involving ‘service users’ in health and social care research, teaching and governance. However, both terms are contested [13]. The general term ‘service user’ has been criticised for the association of the word ‘user’ with illegal drug use, for presenting people as ‘passive’, and for focusing on their use of services rather than other aspects of their identity [14]. There is also no universal definition of ‘involvement’ [15] and the term ‘user-involvement’ may therefore be invoked to describe a range of more of less participatory and integrated practices [15, 16]. Terminology becomes even more complex when comparing over borders [17]. In Denmark, the concept ‘patient involvement’ [patientinddragelse] is generally used to refer to the involvement of people in health research and service improvement, and the people involved most often simply referred to as ‘patients’. The term ‘patient’ however suffers from similar issues as ‘service user’, as it focuses on one particular element of people’s identity. Acknowledging some of these difficulties, and to highlight the active involvement of the people involved, we refer to the people involved in the Empowerment project as ‘co-researchers’ [medforskere] even though their roles and tasks varied considerably within the project. The term ‘user-involvement’ is applied when discussing the process of involving people more generally (i.e. in relation to the international literature) and ‘patient-involvement’, when referring to the discussions within the co-researcher group, as this was the concept they generally used. However, we acknowledge that all terms have certain limitations.

## Methods

The Empowerment project was one of the first in Denmark to formally involve service users, and therefore the process of documenting and describing user-involvement was a key element in the study. The co-researcher group was made aware of this element and knew that not only were they involved in the study as co-researchers, they would also be asked to evaluate their experiences and be the subject of analysis. The following description of our methods begins with a presentation of the involvement of co-researchers in the study, followed by a description of the methods applied in the meeting, from which the findings and recommendations discussed later in the paper derive.

### Methods for user involvement in the project

The empowerment project adopted a consultative and collaborative approach to user involvement [18]. The project was initially devised by the two lead researchers; a psychologist with specialism in psychosocial cancer research and PROM development, and an anthropologist with expertise in the area of user-involvement, but co-researchers and academic colleagues were consulted from a very early stage. Before the submission of the proposal, a group of former and current cancer patients and carers were invited to a workshop to discuss the proposal and its relevance and feasibility. After funding had been obtained, a steering group was set up consisting of the two lead researchers, all co-applicants on the project, and two co-researchers. Half way through the project, one of the co-researchers withdrew from the group and an additional academic researcher, who had joined the project, was added. The steering group has had regular meetings for the duration of the project, and has been a forum for update, discussion and evaluation of the project.

In addition, a group of co-researchers (initially nine current or former cancer patients and one relative) was set up. The group was recruited via various patient groups under the Danish Cancer Society and the Danish Knowledge Centre for User Involvement in Health (VIBIS). No criteria for participation were applied, except for having had experience of cancer either personally or as a relative and having an interest in research. The group met for the first time in May 2015, where they completed a two-day training course, facilitated by two of the lead researchers on the project. This training course included material on what it means to be a co-researcher, the different roles and responsibilities, research methods and approaches and ethics. As part of the course we discussed the conceptualisation of empowerment, various drafts of the research documents, and beginning ideas of what to include in the qualitative interview guide. Another training day was facilitated in August 2015, where participants were trained specifically in interview techniques and had another chance to comment on and revise the interview guide.

After two years, six people had stopped participating in the group and together with the remaining co-researchers it was decided to recruit additional members. A call was posted on the website of some of the patient groups of the Danish Cancer Society and in this round of recruitment the criteria for involvement were extended to having to live locally and having had personal experiences with cancer. The group was also selected based on gender and diagnosis to ensure diversity in the group. Phone interviews were conducted with all applicants before selection, and following this, five additional members were recruited to the group. Shortly after, they participated in an induction day where issues of ethics, confidentiality, roles and responsibilities were discussed.

By the time of writing this paper, a total of 13 former or current cancer patients and one relative have been involved in the project. Ten of them have been women and four of them men, and the majority have come from the Copenhagen area. No ethnic minorities have been represented, a point which will be discussed in more detail below.

The Empowerment project has so far consisted of three main stages: first, an *inception stage* where two qualitative systematic literature reviews were carried out on empowerment amongst cancer patients [19] and existing questionnaires targeting empowerment [20]. Three co-researchers were involved in this stage as reviewers of literature and co-authors on papers.

Second, a *qualitative stage* where 18 semi-structured interviews were conducted with 16 cancer patients in follow up over a period of 6 months. The co-researcher group took part in refining the interview guide and the documents used for the qualitative stage (information leaflets and informed consent form). Five co-researchers were involved as peer interviewers in 11 of the qualitative interviews (for a detailed discussion of this element of the study and the impact of having peer interviewers, see [10]). The co-researchers also participated in a meeting to discuss data analysis in qualitative interviews.

Finally, a *PROM development stage*, in which the qualitative data was used to develop a PROM questionnaire, which will be used in a nation-wide study on empowerment amongst Danish cancer patients in follow up. So far, eight co-researchers have been involved in five meetings and workshops with the aim of developing and testing the PROM and have given feedback on drafts.

In addition to these activities, the group has had several meetings where particular elements of the study have been discussed, e.g. the analysis of the impact of having peer interviewers in the project which later lead to a separate publication on this topic [10]. Until now two co-researchers have co-presented with the lead academic researcher at two separate conferences/meetings. Finally, two and a half years into the project, a meeting was set up between co-researchers and academic researchers to discuss their respective experiences of involvement in the project and to develop a set of recommendations for the further development of user-involvement in the Danish context Table 1.

**Table 1:** Chronological time line of all user involving activities in the Empowerment Project (Jan 2015-Jan 2018)

August 2014	Focus group discussing the proposal
February 2015	Recruitment of co-researchers
April 2015	Two day introduction and training course
May/June 2015	Correspondence with co-researchers around role descriptions, the concept of empowerment and web-page development (following on from discussions had at the training course)
August 2015	One day interview training and discussion of interview guide for the qualitative interviews
April 2015–April 2016	Systematic literature review [19]. Two co-researchers participated in the selection of papers and co-authored journal article
September 2015–March 2016	Qualitative interviews, eleven of them with co-researchers as peer interviewers
March 2016	PI and co-researcher co-presented at conference
March 2016	Workshop where coding and analysis of interviews was discussed
June 2016	Workshop with initial qualitative analysis
March 2016 – December 2016	Systematic literature review [20]. One co-researcher co-authored the paper
July 2016	First analysis of qualitative data was discussed with co-researchers via email correspondence
October 2016	PI and Co-researcher co-presented at conference
November 2016	Workshop about preliminary findings for journal article [10]
April 2017 – December 2017	Writing of journal article with one co-researcher co-authoring the paper
January–February 2017	Recruitment of five new co-researchers
February 2017	Workshop about the development of the questionnaire
March 2017	Introduction day for new co-researcher group
April 2017	Workshop about the development of the questionnaire
June 2017	Workshop about the development of the questionnaire and potential supplementing questionnaire, discussion of recruitment for survey
September 2017	Workshop with academic researchers and co-researchers, discussing the experiences of involvement in the project and beginning recommendations for user-involvement in Denmark
October 2017	Summary of workshop and draft recommendations sent out to all co-researchers and academic researchers for feedback and input
January 2018	Workshop about questionnaire

### Methods for the focus groups and workshop forming the data for this publication

All co-researchers (*n* = 8) and academic researchers (*n* = 7) on the project were invited to attend a four-hour workshop in September 2017, and five co-researchers and four academic researchers were able to participate. The day consisted of a general introduction, followed by two parallel focus groups, where co-researchers and academic researchers were separated, and a final joint workshop to summarize and exchange ideas.

Each of the separate focus groups were asked the same set of questions, (for a full outline of the meeting, including the questions discussed, see Appendix) and the two groups were asked to write down their thoughts on yellow post-it notes, which were subsequently put up on two large pieces of papers and brought back to the joint meeting room. Here the post-it notes were grouped and discussed with input from all participants. This method allowed people to comment on their own or other people’s post-it notes without becoming too ‘personal’, to get ideas from each other, and to provide recommendations based on what was identified as reoccurring themes in the notes.

The focus group with the co-researchers was facilitated by an staff member, external to the project, as we were concerned that the co-researchers would otherwise avoid potential critical comments. One of the researchers on the project conducted the focus group for the academic researcher group and the second joint workshop. This member of the team was selected due to her experience in conducting similar workshops, and because she, as a researcher located abroad, was not part of the day to day management of the project and regular communication with the co-researchers. Both focus groups and the following workshop were recorded, transcribed and analysed with the use of a thematic framework. A summary of the workshop and draft recommendations developed from the findings were sent out to all partners in the project (anonymized) for feedback and comments. Following this, the analysis and recommendations were further refined.

#### Findings

The thematic framework used to analyse the data centred around four key areas: 1) why involvement? 2) when involvement? 3) how involvement? (including activities, payment, training, recruitment and terminology) and 4) impact beyond the project. The analytical process and the findings, including quotes from the workshop and recommendations for involvement, are illustrated in Table 2.

**Table 2:** Themes and recommendations derived from the analysis of workshop with co-researchers and academic researchers with selected quotes^a^

Key question	Co-researchers (CR)	Academic researchers (AR)	Joint discussion	Findings
Why Patient involvement?	I thought that those who don’t have the same resources I have would really benefit from being asked – what are you actually thinking? Is there anything worrying you?	I thought it could help keep ‘a hook’ into the complex world of emotions, conflicting interests and needs, that fluctuate.	We hope it can benefit those that cannot or do not have the resources to get involved in their own treatment (CR).	Proactive and reactive reasons for getting involved
				Increased appreciation for team work
	I believe I can contribute with something. I have had a lot of bad experiences. I have also had some good experiences	It feels like every time I have co-researchers involved, that I am sort of forced to go back to my data. I can’t just say ‘I’ve collected all my data, now I can do the rest behind my desk’	You are forced to doubt (AR).	Ensuring patient-centred research
			Having ‘a hook’ into reality (AR).	
	Bad experiences have also been part of my motivation. I want to do a good job, but it [decisions to get involved in the project] was also [because of] frustrations about some of the experiences I have had		Focusing on patients and continuously revisiting data (AR).	
		Some of what is also really beneficial is that we have to translate, explain and justify why we do things. You are made to be very reflective.		
		Patients contribute something special that doctors or nurses don’t. Especially in this type of research where we are trying to figure out what is important for patients or find out something about the patients. They validate it a bit more…they are just a bit closer to knowing whether we’ve got something.		
	I hope we can help people get a better treatment than we have had			
	The reason you join this kind of thing is to protect the weakest in society.			
	I expect that it would lead to a better definition of patient involvement in research			
When Involvement?	If it is too far from ‘the floor’, e.g. something about medicine, it can be difficult to utilise your experiences.	Patient Involvement should be incorporated into projects if they include the patient perspective.	I think people should involve patients, if it makes sense. Patients should only be involved if it is meaningful. It shouldn’t be [a topic] too far removed from what it involves to be a patient (CR).	Recommendation 1: In every individual project it should be considered whether it is meaningful to involve service users, and researchers should be asked to explicitly explain their reasons for involving or not involving co-researchers in their projects
		You have to consider how it makes sense and there might be different ways and stages in which it can contribute.		
	If it is a study on operative techniques, it might be relevant and it might not, it depends on the method.			
			If it becomes mandatory, you push a few more people ‘over the line’. On the other hand, I have seen cases where there were patients involved, but no one was interested in them (AR).	
		Also depends on the size of the project. A project needs to have a certain size for it to make sense to formalise patient involvement.		Recommendation 2: When involving service users in projects it should be considered how they may be meaningfully involved, and if possible, they should be involved from the beginning, to make involvement as meaningful as possible.
	It has to make sense			
	You have to feel that you can contribute			
	I think everyone should involve patients. It is our bodies after all.	If it isn’t mandatory, there won’t be as many people who can handle or dare to do patient involvement. It probably needs a bit of pressure, but how mandatory? It is not good to ‘push it’ over the head of every researcher.		
			All projects should argue why or why not they have people involved. Should not figure just as ‘icing on the cake’ (CR)	
	I know that you involve patients in other countries and I don’t know why you don’t have to in Denmark.			
	It is best if the patient perspective is included from the beginning.			
How involvement?	Interesting conversations around the questionnaire. We agreed a long part of the way. Made good sense and was a really good day.	One of the things that has surprised me the most is how much it depends on my own abilities. Do I know how to frame what I want from them and help them get involved? I make many decisions about when to involve them. It is about finding out what sort of decisions it makes sense to involve people in.	Uncertainty – do we involve you enough? Sometimes you can get the feeling that it is going a bit slow – but that leads to an important transparency (AR)	Recommendation 3: Researchers need to be reflective and transparent about the desired outcomes of their projects and the role of the co-researchers in reaching those outcomes, but also acknowledge (and be open) about the possibility that initial plans may change and develop and find appropriate ways to communicate that.
*Experiences of involvement activities*				
	Being part of the interviews crossed some boundaries and was very enriching.			
			We have involved more aspects than I had imagined (CR)	
	It is important to have a good researcher with you in that situation (when interviewing).			
		We have tried to inform the co-researchers of the process and keep them up to date.		
	You might go from being an interviewer to being an advisor.		When the project was developed we didn’t know it would be so much [focus on user-involvement] (AC)	
		A lot happens between meetings.		
	Gained a lot from the data analysis. We saw each other’s perspectives on diverse interviews. Fantastic.	I feel a lot of responsibility towards the group.		
		I think I am more worried about whether they are allowed to contribute as much as possible.		
*Payment*	I hadn’t even considered that it should be paid	Most co-researchers have not wanted to be paid, but some did.	It should be free of cost for patients to be involved. The question is whether they should be paid (AR).	Recommendation 4: Researchers need to carefully consider how service users are remunerated for their involvement in a project, at least making it cost-free for people to participate, but also considering people’s individual situation. Alternatives to direct payments may be developed to thank people for their time.
		I have been convinced that you shouldn’t pay because if you do, it involves a commitment.		
	It should be free of cost and not so onerous to be paid [for transport].			
	I think it is difficult. Because then [if paid] it becomes sort of professional.		It might be better if patients were invited to participate in something as remuneration, e.g. an end seminar (CR).	
		For me, it is primarily the administration which makes me want to give up (paying people). Some people are also on benefits. I wouldn’t mind giving them something [else].		
	I think that if it involves a certain amount of hours every week, it has to be paid, but as long as it is just a few meetings here and there, it shouldn’t.			
				Recommendation 5: There needs to be better guidance and organisational support in how to reimburse users for their time.
	I would like, for example, to be invited to a seminar [instead of being paid].			
*Training*	The training didn’t make sense. We don’t need to be ‘mini-researchers’	Do you lose something by training people? I understand that you gain something, but do you also lose something else. The professional patient. Do you begin to see things in system and reduced if you are on a panel and trained.	It might not have been necessary to train the co-research group because we don’t need knowledge about methods and reflexivity and generalisation and subjectivity (CR).	Recommendation 6: Each project should include a training day for researchers and co-researchers where they can get to know each other, agree on an ethical codex for the project and discuss their respective roles and responsibilities.
	It was good for getting to know each other, but we shouldn’t be trained to be small researchers, because that isn’t what we need.			
			No need for ‘mini-researchers’ (CR)	
		I think both groups (patients and academic researchers) should be prepared. There must be some good tools for facilitation, e.g. what are the good questions to pose? Can you become better in that? The point is that you don’t always know what you will get out of it.		
			It is easy for us to say that it is not necessary, but it might be for others… (CR)	Recommendation 7: Besides this, training programmes and initiatives need to be carefully considered in the context of a given project, its individual co-researchers, and the skills of the researchers facilitating the project
	You shouldn’t educate patients in theoretical issues. You should train them to contribute with new things, not to think into the old paradigms – that is the challenge			
			Training needs to focus on what the patient perspective is. (CR)	
			People need an ‘ethical codex’ (CR)	
			You have to be able to understand the essence of the project. (CR)	
			… and your role. (CR)	
			We have discussed that we could use more training in facilitation techniques (AR).	
*Recruitment*	Depends on the project if it needs to be about someone with a particular illness or more broad.	Maybe you could have a ‘structure’ e.g. a panel, which could be involved if it was smaller activities.	There have to be some criteria for recruitment (CR)	Recommendation 8: Recruitment and selection of co-researchers should be considered in every project in relation to its topic and aim, and with a view to ensure as diverse a group as possible.
	Probably best [to recruit] through patient organisations. Not through doctors.			
		In the recruitment for the project we started out by saying that people needed to have an interest. We discussed the purpose of the project, so they didn’t think they were going to change all sorts of things in practice.		
			It shouldn’t be doctors who chose. You need to ask the patient organisations (CR).	
				Recommendation 9: Research institutions are advised to set up an organisation to support user-involvement and a panel which might be involved in smaller activities. This would require a thorough discussion of how diversity can be ensured within such a panel.
			Do you dare to recruit via Facebook? (AR)	
		We strived to have diversity in terms of gender. And we also looked at diagnosis. We also discussed age a little bit and how far diagnosis had to be.		
			Facebook is genius in that respect. Most patient organisations have a Facebook page (CR)	
		You need to be able to work together		
		Is it particular types [of people] who get involved?		
			Lack of guidelines for researchers on recruitment (AR).	
			Researchers need to be less ‘scared’ of the patients (AR).	
*Terminology*			I like [the term] co-researcher but does it imply that it is participatory action research? (AC)	Recommendation 10: It is recommended that the discussion around terminology of user-involvement is continued in the Danish context, to reflect the different roles and identities of patients.
			You shouldn’t signal equality (CR)	
			In journalism you would call it ‘experts of consequence’ (CR)	
			‘Researcher’ sounds wrong in my ears (CR).	
			Could you call it ‘patient consultant’? (CR)	
			I would feel more involved by being called a ‘patient consultant’ than ‘patient co-researcher’ because I don’t do any research (CR).	
			It would be good if it was possible to find a good Danish word to cover this role (AR).	
Impact outside or beyond the project	I mentioned it when I went to follow up and they said they had heard about it. That was fantastic.	Professionally, I have gained a lot of respect for cooperation and involving people. You have to make sure it is not just an empty work. It is more of an art. I am closer to being able to do better next time.		Recommendation 11: Danish researchers need more organisational support and clearer guidelines around the practical sides of user-involvement and it is recommended that a manual for user-involvement is developed in the Danish context, so that more researchers would feel able to involve service users in their projects.
	I haven’t mentioned it in follow up, but I have said it to my GP. Talked to a lot of friends and family about it, and they think it sounds fantastic. You can motivate those of your friends who suffer from serious illness to ask more questions.			
		I think it has made the project more interesting and that it makes my work more interesting, because I am constantly reminded that it actually has significance for people.		
	I have just told it [to people]. For me it is important because I think it might have implications for my own profession.			
		I still don’t feel completely sure how to handle it. I think it would be so good if there were some guidelines.		

^a^All data was collected and analysed in Danish. The quotes included in this table have been translated by the first author of this paper when writing the manuscript

### Why user-involvement?

When discussing reasons for becoming involved in the Empowerment project, the co-researcher group agreed that their most important motivation had been a wish to make a difference for future cancer patients and increase awareness of some of the shortfalls that they themselves had experienced in their contact with the health care system. In line with other studies showing that patients generally get involved in research to put their (good and bad) experiences to use and make a difference for others [21, 22], the motivations of the co-researchers were thus both reactive (motivated by negative experiences) and proactive (wanting to make a change). Some of the co-researchers also acknowledged that they themselves had experienced benefits, for example by acquiring knowledge that they could use in other contexts. In addition, it was hoped that the documentation and evaluation of the project could help develop the practice of user-involvement itself in the Danish context.

The main motivation of the academic researcher group for wanting to involve patients in research was that they believed it would help maintain a connection to the ‘real world’ of the people being studied. Other studies have shown that researchers, who involve service users have gained new knowledge, changed their preferences, increased their skills in communicating with lay audience and changed their attitude to involvement [23, 24]. The academic research team in the Empowerment study was generally positive towards user-involvement from the onset and therefore did not particularly change their attitudes. However, they still acknowledged that working with the co-researcher group had significantly increased their respect for team work and their focus on ensuring that research is patient-centred.

### When user-involvement?

The co-researchers and academic researchers participating in the workshop agreed that projects which explore the experiences, knowledge and perspectives of patients should by principle involve patients. However, they were not certain that involvement should be a requirement for funding, as seen for example in the UK. The academic researcher group argued that if involvement was mandatory, it might encourage more researchers to involve patients and this could be positive, but also potentially lead to issues of tokenism, as discussed extensively in the international literature e.g. [25, 26]. In the joint group there were concerns that if user-involvement were to become mandatory in the Danish context, it might result in less meaningful involvement, because co-researchers would be involved without a clear purpose and ‘just for the sake of it’. It was agreed that it made more sense to involve patients in some projects than others and that a lot depended on the methods of a given project. Therefore, it was recommended that instead of making user-involvement mandatory, all research proposals should include consideration and explanation as to why or why not patients would be involved (Recommendation 1).

Acknowledging the time and logistics spent on involving people in a research process [2], it was also discussed whether projects should have a certain size for them to set up a formalised process of patient involvement. Similar to the principles set down by INVOLVE [4], it was furthermore argued that in projects that involve service users, involvement should happen from the beginning (Recommendation 2).

### How user-involvement?

The individual co-researchers had been involved in the Empowerment project for different lengths of time and had participated in different involvement activities. In their discussion of involvement, they mainly focused on three key activities: 1) the PROM questionnaire, which all of the focus group participants had been involved in developing and giving feedback on and had found both important and relevant; 2) The qualitative interviews, where two of them had been involved as peer interviewers and found that having an academic researcher present at the interview was essential to provide support and keep the interview ‘on track’; 3) The qualitative data analysis, where one co-researcher commented that involvement had enabled the identification of different perspectives.

The group also discussed the general communication between co-researchers and academic researchers and in particular the phone conversations between the academic contact person and the co-researchers prior to recruitment. This was mentioned as important for clarifying the aims and expectations of the project, as well as the activities that the co-researchers were going to participate in. The importance of transparency from the beginning of a project about the expected role and input from service users has been recognized elsewhere [4, 27]. However, there was some sense in the co-researcher group that the aims of the project had changed slightly, and that more attention had been paid to the reflection and analysis of the user-involving element of the project than first anticipated. Based on this, it becomes relevant to add that in any project, researchers and co-researchers should discuss their expectations and the possibility of changing priorities and that guidelines furthermore need to emphasise transparency not only in the beginning, but continuously throughout a project (Recommendation 3).

The academic researcher group focused more generally on the practicalities of involvement. The need for good facilitation skills was mentioned as key to making involvement activities work. In addition, the group raised questions about the extent to which patients should be involved in decisions taken throughout the project. It was acknowledged that communication and up-dates were an important part of involvement in the project. However, as in any other project, many day-to-day decisions had to be made in between meetings and email conversations. One academic researcher felt that it was a big responsibility to decide how much the co-researchers should be consulted to ensure that they were sufficiently, but not overwhelmingly involved.

### Payment/remuneration

Whereas in the UK, it is generally recommended to pay service users for their involvement [1], in the workshop it was agreed, that while involvement should be cost-free for service users, actual payments might shift the role of the co-researcher from being a volunteer to having a different role, perhaps requiring too much commitment and responsibility. It was suggested that rather than paying people for their time, remuneration could perhaps be made in the form of an end seminar or another event towards the end of the project. However, not all co-researchers were present at the workshop and these views may not have been shared by everyone in the larger group. People’s financial situation is a potentially personal and sensitive issue, and if part of the group was to disagree with the majority on payments, it is therefore not certain that they would have made it explicit. From a diversity perspective, it is furthermore important that different segments of society are represented in user-involvement activities, and while lack of payments may be preferably to some, it might also (unintendedly) exclude others. It is therefore of key importance that researchers do not take for granted any particular preferences with regards to payment and carefully consider how decisions around payment can help make their research as inclusive as possible (Recommendation 4).

The logistics around payments and the difficulties in paying transportation costs were identified as a source of significant frustration both from the co-researcher group and the academic researcher, who had been in charge of the administration of the project, and a need for more organisational guidance and support was expressed (Recommendation 5).

### Training

Two focus group participants had participated in the two-day initial training and while they recognized that this has been a good opportunity for getting to know each other, the material had, in their opinion, not been entirely appropriate. Training for involvement is a contentious issue [28–30] and as noted by others, it is important to value the strengths and abilities of co-researchers and prevent their contributions from becoming over professionalised [31]. Similar issues were discussed in our workshop, where it was felt that the content of the training had focused too much on research theories and approaches, rather than roles and responsibilities. However, only two participants at our meeting had participated in the training, and it was mentioned that other participants might have gained from the training, a point which highlights the importance of recognizing diversity within co-researcher groups [6].

With regards to training, the academic researcher group predominantly discussed their own training needs. Reflecting the point made earlier about the importance of facilitation techniques for making involvement activities work, it was agreed that facilitation training would be useful for researchers wanting to involve people in their projects, emphasising that training should not only be considered in relation to co-researchers [6] (Recommendation 6 and 7).

### Recruitment

Discussing recruitment in general, it was agreed that the topic of a given project should dictate the type of people recruited and that some projects might need more specific user groups (e.g. with a particular type of illness) than others. It was also acknowledged that people may have different perspectives, experiences and emotions depending on where they are in their illness journey and that this should be considered when recruiting. On the practical level, the co-researcher group agreed that it was best that people were recruited through patient organisations, rather than for example, through their doctors, as doctors might select particular types of individuals.

The academic researcher group also discussed various strategies for recruitment and selection. In the recruitment for the Empowerment study, diversity had been attempted in the selection of co-researchers in relation to gender and illness, but no minority ethnic patients had expressed an interest in the project, and all selected co-researchers were of Danish origin. 13% of the Danish population are defined as immigrants or descendants of immigrants [32] and their experiences of health may vary significantly from the majority population [33]. Not having any minority ethnic Danes involved in the project may therefore be seen as a limitation. Inclusion, diversity and equal opportunity is a recurring issue in user-involvement [34, 35] and the lack of recruitment from marginalised groups is a general problem [36]. Acknowledging the general issue of diversity in patient-involvement, the groups discussed whether broader recruitment strategies, e.g. via social media platforms such as Facebook, might be useful in reaching a wider audience. However, the academic researchers felt that it was difficult to foresee any potential ethical issues if using Facebook for recruitment and that more guidance from research institutions was needed in order to fully embrace this strategy.

Recruitment was furthermore mentioned as a potential issue for smaller projects, where there may not be time for a thorough and lengthy recruitment process which allows for a diverse group to become involved. Having a strong organisational framework may be a way to help smaller projects with the recruitment process and may also strengthen diversity in recruitment [28]. This supports the case for stronger organisation structures around user-involvement in Denmark. However, the current lack of organisational support in the Danish context (and possibly in other countries as well), makes it important that researchers carefully consider how they recruit their participants, e.g. via patient organisations or broader platforms, such as Facebook to ensure representation of a diverse group (Recommendation 8 and 9).

### Terminology

The terminology around user-involvement is contested and varies internationally. In the workshop, it was discussed whether ‘co-researcher’ was the best term to describe the role they had in the project. On the one hand, it was argued that the term might signal more equality in the work carried out by the academic researchers and the co-researchers than was actually the case. On the other, it was difficult to find alternatives. Other concepts suggested were ‘patient-consultants’ and ‘experts of consequences’, similar to the related concept ‘expert of experience’ [37]. No conclusions were reached on the most appropriate terminology, but it was agreed that it would be useful to have a *‘good Danish word’* (academic researcher) and that terminology has both symbolic and practical value. Any chosen term needs to cover the main roles of patients in research, and since this might vary from project to project it can be difficult to find a common terminology. Recognizing the novelty of user-involvement in Denmark, the Empowerment project was seen as able to make precedence on the words used in the area, and it was therefore agreed to continue the discussion on terminology (Recommendation 10).

### Impact outside or beyond the project

For both co-researchers and academic researchers, involvement in the Empowerment project was reported as having had some impact beyond the project itself. Some of the co-researchers had chosen not to mention their involvement to their doctors, but others had talked with their GPs, family or friends, and experienced positive reactions. One mentioned that talking about involvement in the project might help you help others, another co-researcher reflected that by sharing her experiences more widely, she might help develop user-involvement within her (health) profession. Discussing the impact of the project, the academic researchers mentioned that working with co-researchers had made their work more interesting and relevant and had increased their respect for cooperation and partnerships. However, from the academic researcher group, one key theme reoccurred: the lack of organisational support, guidelines and examples of good practice in the Danish context. This was mentioned in relation to recruitment and the lack of guidelines as to where people could be recruited from, but also in relation to more practical issues, including, how to involve service users early on (e.g. on funding proposals before money has been secured), how to pay them or cover their cost of transport, and what type of activities that service users can most usefully be involved in. It was also mentioned that smaller projects might struggle with the logistics of formalised user-involvement. A final and particular recommendation for the Danish context would therefore be to extend already existing national organisations (such as VIBIS) and develop local organisations and guidelines within universities or research organisations to help with recruitment, practical advice, streamline procedures and support researchers in developing involvement activities (Recommendation 11).

## Conclusion

This paper has discussed the process of involving former and current cancer patients in a Danish research project on the empowerment of cancer patients in follow up. Through a detailed account of the methods used and a reflective and critical analysis of the experiences of both co-researchers and academic researchers, we have aimed to add to the current very limited knowledge base on user-involvement in the Danish context. In addition, we have provided a set of early recommendations for the further development of the practice of user-involvement in Danish Health Research.

The experiences of academic researchers and co-researchers in the Empowerment study mirror many of the issues encountered in the international literature. There thus seem to be good reason for cross-national discussions and exchange of ideas regarding how such challenges can be overcome. The lack of Danish guidelines and organisational support described in the workshop is a particular example of an area where Denmark could learn from other countries and take on board international experiences of developing organisational structures to support user-involvement.

However, the Empowerment study has also illustrated that user-involvement is contextual, and that some principles and practices may not translate directly. The way user-involvement is conceptualised and fits within existing organisational structures and practices varies from locality to locality, and when developing involvement activities, researchers thus need to carefully consider the particular local context in which they are working, alongside any international guidelines and examples of good practice.

### Additional file

Additional file 1: GRIPP2 short form for the Empowerment Study. (DOCX 15 kb)

## Appendix

### Outline of workshop 28 September 2017

12–12.15: Introduction and presentation of workshop (whole group).

12.15–13.15: Dividing the two groups into two separate focus groups: 1) co-researcher and 2) academic researchers.

Questions for discussion in the focus groups:

1. Why did you choose to become involved in the project? What caught your interest?
2. What were your expectations of the project/expectations of your own involvement in the project?
3. What is your general opinion about involving patients or carers in research? What is most important in the cooperation between co-researchers and academic researchers? Are some projects more suitable for involvement than others?
4. How have you found the process of involvement in the Empowerment project?
  - Questions can include:
    i. The way people have been recruited – how you think people should be recruited? Are any particular criteria needed?
    ii. Payment – should people be paid? If so, for all or only some activities? What type of payment?
    iii. Training – how did you find the training? Should patients be training for involvement? Should academics be trained?
    iv. How did involvement work in the different stages?
    v. How did the communication work?
    vi. Did you feel that you knew what the role of co-researcher involved? (only co-researchers)
    vii. Did you experience any challenges in your work with academic researchers/co-researchers? If so, how were they/how could they have been dealt with?
5. Has your involvement in the project had any impact on you (personally and/or professionally)?

13.30–13.45: Summary of main points from the groups – written on post-it notes and attached to flip chart paper before lunch.

13.45–14.00: Break/Lunch.

14.00–15.30: Creative process:

- Academic researchers and co-researchers discuss the post-it notes together and group them according to theme and importance.

15.30–16.00: Conclusions and agreement on further actions.

## Abbreviations

PPI: Patient and Public Involvement
PROM: Patient Reported Outcome Measure

## References

[1] McKeown M, Malihi-Shoja L, Downe S (2010). The Comensus writing collective. Service user and Carer involvement in higher education. Service user and Carer involvement in education for health and social care.

[2] McLaughlin H (2009). Service-user research in health and social care.

[3] Boote J, Wong R, Booth A (2015). ‘Talking the talk or walking the walk?’ A bibliometric review of the literature on public involvement in health research published between 1995 and 2009. Health Expect.

[4] INVOLVE (2015). Public involvement in research: values and principles framework October.

[5] INVOLVE (2012). Developing training and support for public involvement in research.

[6] INVOLVE (2012). Diversity and inclusion: What’s it about and why is it important for public involvement in research?.

[7] VIBIS. Om VIBIS . n.d. [cited 2018 May 3]. Available from: https://danskepatienter.dk/vibis/om-vibis

[8] Kjær Jørgensen BM. Patienter skal inddrages mere i forskningen (patients should be more involved in research). Ny Viden 9th ed. 2017:12–4.

[9] Johnsen AT, Eskildsen NB, Thomsen TG, Groenvold M, Ross L, Jørgensen CR. Conceptualising patient empowerment in cancer follow-up by combining theory and qualitative data. Acta Oncol. 2016;56(2):232–8. 10.1080/0284186X.2016.1267403 28080181

[10] Jørgensen CR, Eskildsen NB, Thomsen TG, Nielsen ID, Johnsen AT. The impact of using peer interviewers in a study of patient empowerment amongst people in cancer follow up. Health Expect. 2018;21(3):20–627. 10.1111/hex.12655 PMC5980595 29206313

[11] Staniszewska S, Brett J, Mockford C, Barber R (2011). The GRIPP checklist: strengthening the quality of patient and public involvement reporting in research. Int J Technol Assess Health Care.

[12] Staniszewska S, Brett J, Simera I, Seers K, Mockford C, Goodlad S, et al. GRIPP2 reporting checklists: tools to improve reporting of patient and public involvement in research. Research Involvement and Engagement . 2017 3 (1). [cited 2018 Mar 2]; Available from: http://researchinvolvement.biomedcentral.com/articles/10.1186/s40900-017-0062-2 10.1186/s40900-017-0062-2 PMC5611595 29062538

[13] Barnes M, Cotterell P. From margin to mainstream. In: Critical perspectives on user involvement. Bristol: Policy Press. p. 2012.

[14] Beresford P, Beresford P, Carr S (2012). The theory and philosophy behind user-involvement. Social Care, Service Users and User Involvement.

[15] Purchell R, Rickard W, Wyatt K, Barnes M, Cotterell P (2012). Should we? Could we? Measuring involvement. Critical perspectives on user involvement.

[16] Fleming J, Beresford P, Carr S (2012). Service user involvement - what it is and what it could be: lessons from the standards we expect project. Social Care, Service Users and User Involvement.

[17] Dent M, Pahor M (2015). Patient involvement in Europe – a comparative framework. Journal of Health Organization and Management.

[18] Beresford P (2007). User involvement, research and health inequalities: developing new directions: user involvement, research and health inequalities. Health & Social Care in the Community.

[19] Jørgensen CR, Thomsen TG, Ross L, Dietz SM, Therkildsen S, Groenvold M, Rasmussen CL, Johnsen AT. What facilitates ’patient empowerment’ in cancer patients during follow-up? – a qualitative systematic review of the literature. Qual Health Res. 2017;28(2):292–304. 10.1177/1049732317721477 28758544

[20] Eskildsen NB, Jørgensen CR, Thomsen TG, Ross L, Dietz SM, Groenvold M, Johnsen AT. Patient empowerment – a systematic review of questionnaires measuring empowerment in cancer patients. Acta Oncol. 2016;56(2):156–65. 10.1080/0284186X.2016.1267402 28077053

[21] Ashcroft J, Wykes T, Taylor J, Crowther A, Szmukler G (2016). Impact on the individual: what do patients and carers gain, lose and expect from being involved in research?. J Ment Health.

[22] Sargeant A, Payne S, Gott M, Small N, Oliviere D (2007). User involvement in palliative care: motivational factors for service users and professionals. Progress in Palliative Care.

[23] Staley K (2017). Changing what researchers “think and do”: is this how involvement impacts on research?. Research for All.

[24] Staley K, Abbey-Vital I, Nolan C. The impact of involvement on researchers: a learning experience. Research Involvement and Engagement. 2017 3(1). [cited 2018 Mar 2]; Available from: http://researchinvolvement.biomedcentral.com/articles/10.1186/s40900-017-0071-1 10.1186/s40900-017-0071-1 PMC5611580 29062545

[25] Ocloo J, Matthews R (2016). From tokenism to empowerment: progressing patient and public involvement in healthcare improvement. BMJ Quality & Safety.

[26] Supple D, Roberts A, Hudson V, Masefield S, Fitch N, Rahmen M, et al. From tokenism to meaningful engagement: best practices in patient involvement in an EU project. Research Involvement and Engagement. 2015 ;1(1). [cited 2018 Mar 2] Available from: http://researchinvolvement.biomedcentral.com/articles/10.1186/s40900-015-0004-9 10.1186/s40900-015-0004-9 PMC5598090 29062494

[27] Howe A, Mathie E, Munday D, Cowe M, Goodman C, Keenan J, et al. Learning to work together – lessons from a reflective analysis of a research project on public involvement. Research Involvement and Engagement. 2017 ;3(1). [cited 2018 Mar 2] Available from: http://researchinvolvement.biomedcentral.com/articles/10.1186/s40900-016-0051-x 10.1186/s40900-016-0051-x PMC5611599 29062526

[28] Jørgensen CR, Purkis J, Blaxter L, Tulip S. Accredited Training for User Involvement in Higher Education Teaching – Exploring an Innovative Training Programme in Public Involvement and Partnership Working. International Journal of Practice-based Learning in Health and Social Care. 2016;4(2):49–62.

[29] Mockford C, Murray M, Seers K, Oyebode J, Grant R, Boex S, et al. A SHARED study-the benefits and costs of setting up a health research study involving lay co-researchers and how we overcame the challenges. Research Involvement and Engagement. 2016 ;2(1). [cited 2017 Sep 8] Available from: http://www.researchinvolvement.com/content/2/1/8 10.1186/s40900-016-0021-3 PMC5611649 29062509

[30] Dudley L, Gamble C, Allam A, Bell P, Buck D, Goodare H, et al. A little more conversation please? Qualitative study of researchers’ and patients’ interview accounts of training for patient and public involvement in clinical trials. Trials 2015 ;16(1). [cited 2016 Dec 5] Available from: http://trialsjournal.biomedcentral.com/articles/10.1186/s13063-015-0667-4 10.1186/s13063-015-0667-4 PMC4410574 25928689

[31] SCIE. At a Glance 19: Building user and carer involvement in Soc Work Educ 2009 Nov; Available from: https://www.scie.org.uk/publications/ataglance/ataglance19.asp

[32] Danmarks Statistik (2017). Indvandrere i Danmark 2017 (Immigrants in Denmark 2017).

[33] Esholdt HF, Fuglsang M, Region Hovedstaden, Enheden for Brugerundersøgelser (2009). Etniske forskelle i patienters oplevelser: en spørgeskema- og interviewundersøgelse om etniske forskelle i patientoplevelser i forløbet fra praktiserende læge til hospital : forsknings- og udviklingsrapport om indvandrere/efterkommere i sundhedsvæsenet.

[34] Branfield F (2009). Developing user involvement in social work education.

[35] Brett J, Staniszewska S, Mockford C, Herron-Marx S, Hughes J, Tysall C (2014). Mapping the impact of patient and public involvement on health and social care research: a systematic review. Health Expect.

[36] Green G. Power to the people: To what extent has public involvement in applied health research achieved this? Research Involvement and Engagement . 2016 ;2 (1). [cited 2018 Mar 2] Available from: http://researchinvolvement.biomedcentral.com/articles/10.1186/s40900-016-0042-y 10.1186/s40900-016-0042-y PMC5831888 29507763

[37] Newbigging K, Roy A, McKeown M, French B, Beresford P, Carr S, Habte-Mariam Z (2012). Involving ethnically diverse service users in the ResearchProcess: alliances and action. Social Care, Service Users and User Involvement.

