# Supplementary material for: User involvement in a Danish project on the empowerment of cancer patients – experiences and early recommendations for further practice
Source: Res Involv Engagem. 2018 Aug 13;4:26. doi: 10.1186/s40900-018-0105-3 (PMC6088427; doi:10.1186/s40900-018-0105-3)
Supplement: Supplementary file 1 — GRIPP2 short form for the Empowerment Study. (DOCX 15 kb) [file 40900_2018_105_MOESM1_ESM.docx]

**Table 2 GRIPP2 short form for the Empowerment Study**

**1: Aim:** The overall aims of the Empowerment project were to explore the experiences of empowerment of cancer patients in follow up and develop a patient reported outcome measurement (PROM) questionnaire to assess levels of empowerment within this group. The main reason for involving service users in the project was that they, through their inputs, would make the project more sensitive and relevant to cancer patients in follow up.

**2: Methods:** A group of 14 former or current cancer patients were involved as co-researchers from the early stages and all through the project. Co-researchers gave feedback on the proposal, helped develop project documents and research tools, acted as peer interviewers in qualitative interviews, participated in data analysis and development of questionnaires, co-authored journal articles and co-presented at conferences. The workshop which forms the basis for this paper consisted of two parallel focus groups (one with co-researchers and one with academic researchers) and a joint group discussion, following an interactive and informal format to facilitate discussion and exchange of ideas. The summary from the workshop was circulated and discussed with the co-researchers.
**3: Study results Outcomes:** This paper reports on the findings from a workshop discussing the experiences of the co-researchers and researcher in the project. Questions of why, when, and how to involve service users in health care research were extensively discussed, alongside issues of terminology and impact beyond the project.

**4: Discussion and conclusions:** Eleven early recommendations were derived from the discussions between co-researchers and academic researchers, including the need for more guidance and organisational support for Danish researchers around user-involvement and the careful consideration of methods for recruitment, training and payment of service users in the Danish context.

**5: Reflections/critical perspective:** Experiences from involving service users in the Empowerment project have been very positive, and as described in the paper, much has been gained from the process. Some of the challenges have been lack of guidelines and support around payment. Recruitment of a diverse group was also found difficult, and in the second round of recruitment it was decided to recruit more locally, as travel became an issue for some co-researchers.
